# Insights into psychological characteristics of persons (not) agreeing to use an e-coach-application to reduce elevated Internet Use Disorder tendencies

**DOI:** 10.1016/j.abrep.2024.100564

**Published:** 2024-09-26

**Authors:** Christian Montag, Jon D. Elhai, Christopher Kannen, Anja Bischof, Dominique Brandt, Hannah Schmidt, Dmitri Rozgonjuk, Hans-Jürgen Rumpf

**Affiliations:** a Department of Molecular Psychology, Institute of Psychology and Education, Ulm University, Ulm, Germany; b Department of Psychology, and Department of Psychiatry, University of Toledo, Toledo, OH, United States; c Department of Psychiatry and Psychotherapy, University of Lübeck, Lübeck, Germany

**Keywords:** Internet Use Disorder, E-coach, Treatment, Therapy, Personality, Fear of missing out

## Abstract

The present work aims to shed light on the question of whether certain psychological characteristics go along with choosing an e-coach offer to support healthy Internet use when reporting elevated Internet Use Disorder (IUD) tendencies. Data were from a large-scale stepped care approach study to treat persons with varying degrees of IUD tendencies. Recruitment for advertising the download of a smartphone app included social media, videos by influencers, paid ads, TV, radio, newspapers, workshops, and vocational schools. We contrasted a final sample of 184 declining, 907 agreeing, and 216 agreeing but not providing follow-up details needed to use such an e-coach, as well as 995 who did not explicitly decline or agree to participate (but refrained from using the e-coach). Participants were compared on several study variables, including sociodemographics, mental health, fear of missing out, personality, perceived stress, and IUD tendencies. Interestingly, we observed only two significant findings when contrasting the aforementioned groups using ANOVA. First, the group not agreeing to use the e-coach was associated with the lowest IUD tendencies and highest conscientiousness scores. Second, agreeing to use the e-coach was associated with older age. Further significant differences could be observed, but in general the accompanying effect sizes were very mild. In sum, the present study findings support the idea that greater subjective burden of IUD might result in more willingness to seek e-help, but also the personality trait of conscientiousness and a person’s age might play a role here.

## Highlights

• Participants agreeing or not agreeing to use an e-coach to reduce Internet Use Disorder (IUD) tendencies was investigated.
• Psychological characteristics of different user groups agreeing or not agreeing to use the e-coach are investigated.
• IUD tendencies, a facet of sofalizing, conscientiousness and age turned out to be relevant variables (to varying degrees).

## 1 Introduction

The global prevalence of Internet Use Disorders (IUDs) has been summarized in a meta-analysis to be around 7 % if generalized IUD measures have been used in studies (Pan et al., 2020). When asking for more specific IUDs such as Internet Gaming Disorder, lower prevalence rates around 2.47 % were observed. The pandemic has led to an increase in Internet Use Disorders (Montag et al., 2024, Paschke et al., 2021, Rozgonjuk et al., 2022, Teng et al., 2021), underlining the relevance of finding treatments for people suffering from IUDs. This said, at the moment still debated is how to assess IUD best and which framework is most fitting. In the present work, we used the CIUS which stands for the Compulsive Internet Use Scale, whereas many of the items of this scale tap into the addiction framework (where we also think IUD theoretically fits best).

The World Psychiatric Association Lancet Psychiatry Commission stressed the benefits of digital interventions to increase access to treatment for mental disorders in the future (Bhugra et al., 2017). Smartphone applications have proven efficacy in mental disorder treatment such as for major depression based on results of a meta-analysis (Firth et al., 2017). Such applications offer interactive engagement with users and are based on therapeutic principles such as cognitive behavioral treatment aimed at facilitating cognitive restructuring (Morello et al., 2023). They have shown potential for effective treatment and cost-saving help (Koh et al., 2022). Given the treatment gap for mental disorders in general (Shidhaye, Lund, & Chisholm, 2015) and the lack of early intervention approaches specifically for IUDs (Rumpf et al., 2018) with few recent exceptions (Bernstein et al., 2023, Boumparis et al., 2023), it is highly relevant to offer accessible, alternative help, which in particular might be helpful when participants are in the early stages of developing psychopathology.

At the same time, uptake of and adherence to digital interventions is a critical factor (Gilbody et al., 2015) and it would be of high relevance to establish evidence on who responds to an e-health offer and who declines. A recent systematic review on digital mental health interventions revealed that user engagement depends on several variables including sociodemographic characteristics, personal traits, mental health status, technology experience and skills, and the level of guidance (Borghouts et al., 2021). However, to the best of our knowledge, data on characteristics of users for the uptake of e-health interventions in the realm of IUDs are currently not available. Further, we also investigate variables such as personality, fear of missing out, and life satisfaction, which are known to play a role in context of Internet Use Disorders. In detail it has been shown that higher neuroticism and lower conscientiousness go along with mobile IUD tendencies (Marengo et al., 2020), and IUD tendencies also go along with lower satisfaction (Lachmann et al., 2016) and higher fear of missing out (Elhai et al., 2020). Beyond this, also constructs such as sofalizing, mental health and perceived stress are analyzed. Sofalizing represents a new construct aiming to describe people preferring to communicate online with their friends (instead of meeting offline) and recent work shed light on associations with IUD (Montag et al., 2024). Furthermore, it has been put forward that IUD might develop from coping with negative emotions or stressful experiences in everyday life. In addition, it has been suggested that a history of mental health issues might represent a vulnerability factor for developing IUDs. Therefore, assessing perceived stress in one’s life and mental health might be of interest to also understand who is more willing to use an e-coach or not.

The present study is the first one aiming to provide individuals with elevated IUD tendencies help to reduce IUD symptoms with a comprehensive e-coach approach. The present analysis does not examine the effectiveness of this intervention, but instead focuses on the relevant question of whether some person characteristics are associated with willingness to take help from an e-coach. As participants of our study first completed a screening questionnaire assessing IUD tendencies alongside several other questionnaires and had the opportunity after the screening to obtain help from an e-coach when crossing a threshold for elevated IUDs levels, the present study design was able to provide an answer to the question raised. As this part of the study was not preregistered (see the full study protocol here: Bischof et al., 2022), the findings presented in this work should be seen as exploratory and need to be replicated.

## 2 Methods

### 2.1 Recruitment process for the present study

All participants downloaded an application called smart@net, where they completed questionnaires providing insights into IUD tendencies, fear of missing out (FoMO), personality, and life satisfaction. As the app was in the German language, it attracted German speaking participants. FoMO, personality and life satisfaction were studied in the realm of this work, because they have been all linked to IUD tendencies in the past (Elhai et al., 2020, Lachmann et al., 2019, Lachmann et al., 2018). As an incentive to fill in these inventories, participants were provided with feedback on their scores compared with the other participants of the study (the comparison of course was carried out with anonymous data). This feedback was updated in real time, and participants could revisit the app at a later time to compare their own data to an even larger dataset. Importantly, the smart@net-application was advertised via social media (including videos by influencers and paid ads), TV, radio, newspapers, online events, talks, and workshops with vocational students in the context of a study testing an online intervention to reduce IUD tendencies by means of a stepped care approach. Hence, every interested participant between 16 and 67 years could download the app, which was advertised via many mass media channels. When reaching a score of 21 or higher on the Compulsive Internet Use Scale (CIUS), participants were invited to use an e-coach to guide them for 28 days with motivation techniques and advice on how to reduce problematic smartphone and IUD tendencies. Components of the app included methods to increase readiness and self-efficacy for behavior change related to key goals of Motivational Interviewing (MI) (Miller & Rollnick, 2012) as well as elements of Cognitive Behavioral Treatment. Participants completed questionnaires and received specific, individualized feedback. Predictors of problematic Internet use, such as FoMO, were monitored and reported. Push notifications were used to invite participants to add new entries. Participants were supported with behavior change plans and recognized for their progress. The study followed a stepped-care approach with telephone counseling and online-therapy as steps 2 and 3 after the app intervention. Potential harm related to the interventions was not expected. Participants had not to refrain from any additional help or psychotherapy once enrolled into the study. Seeking help after or during the intervention could have been triggered by the intervention and was considered welcome. Nevertheless, in step 2 and step 3, the occurrence of any adverse events was assessed. The detailed study protocol has been published previously (Bischof et al., 2022). The study was approved by the local ethic committee at University of Lübeck, Germany. Please note that some of the here presented data overlap with a paper on well-being and Internet Use Disorders published recently (Montag et al., 2024).

In total, for the present study data from N=6,619 participants could be collected (exclusion criteria for being enrolled were not using a smartphone, psychotherapy in the last four weeks prior to enrollment and German language proficiency). N=3,819 were excluded because they did not reach a score of 21 or higher. We only analyzed those participants who were offered the intervention and either declined (n = 231), agreed (n = 967), agreed but did not provide follow-up details necessary to participate (n = 275; such as providing consent for the intervention), or neither explicitly declined or agreed (n = 1,608). We further excluded those who did not take the optional part of the survey, including personality and FoMO measures, resulting in 194, 919, and 227, and 1,028 participants, respectively. We excluded participants who responded carelessly (see below), resulting in a sample of 190, 919, 223, and 1,017 participants, respectively. We also excluded participants who did not endorse male or female sex (resulting in 184, 907, 216, and 995 participants, respectively) because we analyzed sex as a two-category variable below. Unfortunately, the number of non-binary participants is too small to run robust statistical analysis on this group.

### 2.2 Questionnaires

The Compulsive Internet Use Scale (Meerkerk et al., 2009) was administered in German language (Gürtler et al., 2015). This scale consists of 14 items that are presented in a five-response format ranging from 0 (never) to 4 (very often). Higher scores indicate higher Internet Use Disorder tendencies. The internal consistencies in the present sample are as follows: α = 0.77 and ω = 0.82. Although not being based initially on the DSM-5 approach, the CIUS showed good performance when validated against DSM-5 criteria in its full form and a short version (Besser et al., 2017).

Satisfaction with life (Diener et al., 1985) was assessed using the German version of Diener’s scale (Janke and Glöckner-Rist, 2012). This scale consists of five items answered in a five-answer format ranging from 1 = ‘does not apply at all’ to 5 = ‘very much applies’. Higher scores indicated higher satisfaction with life. Internal consistencies in the present sample were as follows: α = 0.83 and ω = 0.85.

The Sofalizing scale (Tosuntaş et al., 2020) was administered in German language (Montag et al., 2024). The scale consists of 11 items; five items assess Online Displacement, and six items assess Social Compensation. The items can be answered in a five-answer format, ranging from 0 (never) to 4 (very often). Higher scores indicate higher Online Displacement or higher Social Compensation. Internal consistencies in the present sample were as follows: α = 0.77 and ω = 0.79 for Online Displacement, and α = 0.75 and ω = 0.82 for Social Compensation.

Perceived stress in the previous month was assessed using the Perceived Stress Scale 4 (PSS-4; Warttig et al., 2013). We used the German version of the PSS-4 (Klein et al., 2016). The scale consists of four items answered in a five-answer format ranging from 1 (never) to 5 (very often). Two items need to be reversed before the scale’s items can be summed. Higher scores indicated higher perceived stress in the last month. Reliability was α = 0.72 and ω = 0.77.

Mental Health was assessed using the five-item Mental Health Inventory (MHI-5; Berwick et al., 1991). The validated German version showed good validity in particular with respect to mood and anxiety disorders (Rumpf et al., 2002). The scale consists of five items with an answer format ranging between 0 (none of the time) to 4 (all of the time). Reliability for the MHI scale was α = 0.58 and ω = 0.67.

### 2.3 Optional questionnaires

Participants could voluntarily fill in additional questionnaires to receive more feedback. Therefore, the following questionnaires were not completed by all the participants (see above data cleaning steps).

FoMO tendencies were assessed using the German version of the FoMO scale (Wegmann et al., 2017). This scale consists of 12 items, of which five items assess a FoMO trait, which is linked to general FoMO. Seven items represent state FoMO, which assesses FoMO tendencies in the online context. Items are answered via a five-answer format ranging from 1 = strongly disagree to 5 = strongly agree. Higher scores for trait and state FoMO represent higher FoMO tendencies. Internal consistencies in the present sample were as follows: α = 0.74 and ω = 0.84 for trait FoMO, and α = 0.77 and ω = 0.85 for state FoMO.

The Big Five of Personality traits were assessed using the IPIP-20 (Donnellan et al., 2006). German items were retrieved from the International Personality Item Pool (IPIP). This scale consists of 20 items assessing five personality traits: Extraversion, Agreeableness, Conscientiousness, Neuroticism and Intellect/Imagination. Each personality trait was assessed using four items with an answer format ranging from 1 (strongly disagree) to 5 (strongly agree). Eight items need to be reversed before each dimension can be summed. Higher scores indicate higher personality expression on the respective scale. Internal consistencies in the present sample were as follows: Extraversion α = 0.77 and ω = 0.78; Agreeableness α = 0.71 and ω = 0.80; Conscientiousness α = 0.60 and ω = 0.64; Neuroticism α = 0.73 and ω = 0.78 and Intellect/Imagination: α = 0.64 and ω = 0.70.

### 2.4 Statistical analyses

In the final analysis, only participants with a CIUS score > 20 were included. Further data cleaning was performed using R 4.30 using the careless package to detect careless responses, and we excluded a few participants who indicated the same response to at least 20 consecutive items. We used the gmodels and psych packages to compute a chi-square test for the association between our primary groups (those agreeing to or declining intervention) and sex. We computed the means and standard deviations for continuous variables and frequencies for sex and groups. Skewness and kurtosis were examined for the normality of the dependent variables (scale scores and age). Alphas were computed using the fmsb package, and omegas were computed using the GPArotation package. Correlations in each group are presented and computed using the corrplot package. One-way ANOVAs (group as the independent variable, with each psychological scale score as a separate dependent variable) were computed using car and sjstats packages, with a correction for multiple statistical tests conducted (discussed below); next, Tukey multiple pairwise between-group comparisons were implemented using the multcomp package. Finally, we computed a multinomial logistic regression analysis using R’s nnet and DescTools packages to examine associations between the psychological scale variables and age (predictors) with the four levels of our group variable (dependent variable); the Not Agreeing group (n = 184) served as the reference group.

## 3 Results

**Fig. 1:**
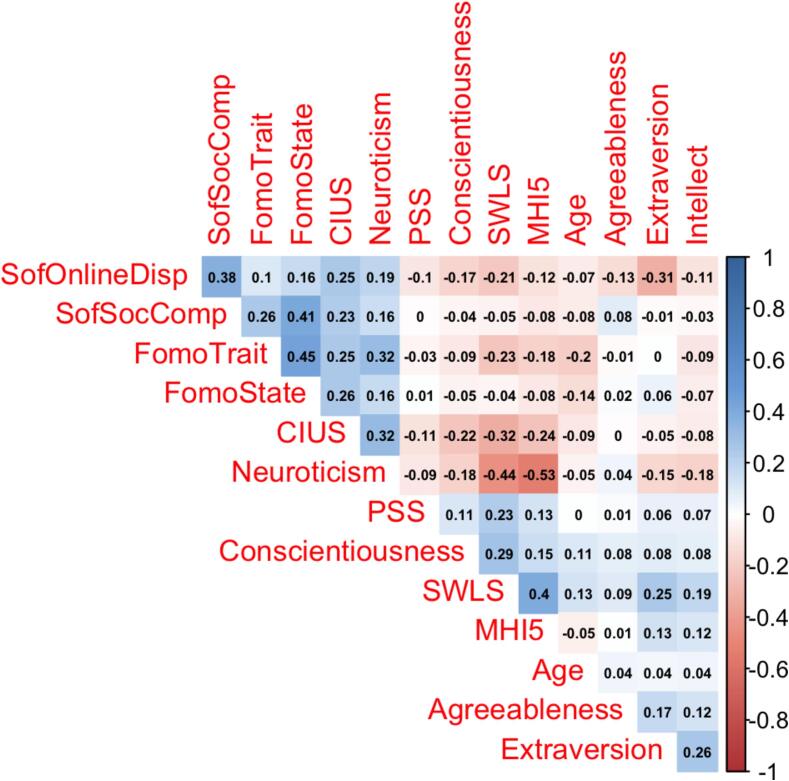
Pearson correlations between variables, for the entire sample. SofSocComp = Sofalizing Social Compensation; SofOnlineDisp = Sofalizing Online Displacement; CIUS=Compulsive Internet Use Scale; PSS=Perceived Stress Scale; SWLS = Satisfaction with Life Scale; MHI5 = Mental Health Inventory-5 Scale. The figure is a correlation heatmap, with correlations displayed, with darker colored cells indicating stronger relationships (blue for positive, and red for inverse relations). (For interpretation of the references to colour in this figure legend, the reader is referred to the web version of this article.)

The sample had 184 participants (92 men, 50 %) declining to participate, 907 participants (including 494 men, 54,47 %) agreeing, 216 participants (111 men; 51,39 %) agreeing to participate in the intervention but not providing required follow up details, and 995 (including 505 men; 50,75 %) neither agreeing or declining to participate (one could perceive this group as passively declining by not providing consent). First, we tested the group by sex association, and found no significant relationship, χ^2^(1) = 3.11, p = 0.375. Neither skewness nor kurtosis was greater than 2 in absolute size for any scale score. All the relevant variables are presented as descriptive statistics in Table 1. These statistics are presented for the complete sample under investigation, agreeing to the intervention subsample, agreeing but not providing follow-up details, not agreeing subsample, and the neither agreeing nor disagreeing sample, compared with ANOVAs in Table 1. To control for Type I error from conducting multiple ANOVAs, we used the Holm correction method (Holm, 1979), which compared to the Bonferroni method decreases the possibility of Type II error as well. This method involves arranging obtained p values in ascending order and first evaluating the smallest p value against an alpha of 0.05/k (where k is the number of tests); subsequent p values are evaluated against a modified alpha of 0.05/k where k indicates the number of remaining tests (e.g., for five remaining tests, 0.05/k-5), and once a non-significant p value is obtained, all larger p values are judged non-significant. Table 1 also displays Tukey pairwise between-group comparisons. Table 1′s p values column demonstrates that seven ANOVAs were statistically significant at p < 0.05, but one dependent variable (MHI-5) would not be significant using the Holm correction by being judged against its modified alpha. Correlations between all variables of interest, including age, are presented in Fig. 1 for the entire samples.

**Table 1:** Means and standard deviations for primary continuous variables separated by group (agreeing vs. not agreeing to the intervention).

**Variable**	**Agreeing** **(n = 907)** **M** **(SD)**	**Not agreeing** **(n = 184)** **M** **(SD)**	**Agreeing but not providing details (n = 216** **M** **(SD)**	**Neither agreeing nor disagreeing (n = 995)** **M** **(SD)**	**F(1,2298)**	**p**	**η^2^_p_**
FoMO Trait	13.76_a_ (4.20)	14.20 (4.12)	14.13 (4.02)	14.52_b_ (4.09)	5.30	**0.001**	0.007
FoMO State	18.69_a_ (5.27)	19.09 (5.15)	19.42 (5.02)	19.89_b_ (5.28)	8.38	**0<.001**	0.01
PSS	12.04 (1.40)	12.11 (1.42)	12.20 (1.49)	12.16 (1.36)	1.50	0.21	0.002
SWLS	15.36 (4.34)	15.67 (4.11)	15.18 (4.25)	15.40 (4.03)	0.49	0.69	0<.001
Sof-Online Displacement	4.37 (3.867)	4.20 (3.81)	4.28 (3.41)	4.19 (3.52)	0.39	0.76	0<.001
Sof-Social Comp	10.45 (4.54)	9.62_a_ (4.44)	10.36 (4.32)	10.84_b_ (4.20)	4.50	**0.003**	0.006
CIUS	30.31_a_ (7.08)	28.88_b_ (6.34)	30.11 (7.44)	29.19_b_ (6.56)	5.38	**0.001**	0.007
Extraversion	8.63 (2.87)	8.51 (2.72)	8.57 (2.92)	8.81 (2.69)	1.11	0.34	0.001
Agreeableness	13.90 (2.11)	13.83 (2.12)	14.14 (2.12)	14.10 (2.07)	2.17	0.09	0.003
Conscientiousness	12.54_a_ (3.01)	13.42_b_ (2.62)	13.13_b_ (2.63)	12.93_b_ (2.71)	7.14	**0<.001**	0.009
Neuroticism	12.91 (3.20)	12.61 (3.01)	12.97 (3.00)	12.93 (3.05)	0.62	0.60	0<.001
Intellect	13.35 (1.82)	13.43 (1.84)	13.23 (1.94)	13.33 (1.85)	2.61	0.05	0.003
MHI-5	9.93_a_ (3.25)	10.33 (3.13)	9.97 (2.99)	10.37_b_ (3.15)	3.48	.02⊕	0.005
Age	29.38_a_ (10.72)	27.49_ab_ (12.58)	26.79_bd_ (10.29)	25.03_d_ (10.53)	25.85	**0<.001**	0.03

Note*.* PSS=Perceived Stress Scale; SWLS=Satisfaction with Life Scale; Sof-Online Displacement = Sofalizing Online Displacement; Sof-Soc Comp = Sofalizing Social Compensation; CIUS=Compulsive Internet Use Scale; SWLS = Satisfaction with Life Scale; MHI-5 = Mental Health Inventory-5 Scale. Group was coded “1″ for “agreeing,” 2 for “not agreeing,” “3” for agreeing but not providing follow-up details, and “4” for neither agreeing nor disagreeing to participate in the intervention. Means within a row that have subscript letters but no subscripts in common indicate significant differences (p < 0.05) between those means using Tukey pairwise comparisons.

⊕ indicates that the dependent variable was not statistically significant when judged against the modified Holm alpha level.

Our multinomial logistic regression analysis included the psychological scale scores and age (from Table 1) as predictors of our four groups (the dependent variable). Full results are available in Supplementary Table S1. In brief, the model was significant and accounted for 9 % of the variance in group membership, χ^2^(3) = 124.86, p < 0.001, Nagelkerke’s *R*^2^ = 0.09. Comparing the group declining the intervention (reference group) to all other groups, only four significant effects (p < 0.01) were found: 1) higher Sofalizing Social Compensation scores were more likely in the Agreeing group, 2) higher Sofalizing Social Compensation scores were more likely in the Neither Agreeing Nor Disagreeing group, 3) lower Conscientiousness scores were more likely in the Agreeing group, and 4) younger age was more likely in the Neither Agreeing Nor Disagreeing group.

## 4 Discussion

The aim of the present work was to understand the individual characteristics of persons who decide to use an e-coach when being afflicted with elevated IUD tendencies versus those who decline. We investigated a range of variables, including sociodemographics, personality, perceived stress, FoMO, satisfaction with life and IUD tendencies, to obtain first insights into this relevant research question. Interestingly, we observed only few differences when taking a look at the myriad variables assessed. Basically, three variables were of interest when taking a closer look. First, those rejecting use of the e-coach also had the lowest IUD scores. Hence, a lower subjective burden is indeed associated with lower likelihood of choosing to use the e-coach. Second, the same participant group was associated with the highest conscientiousness scores (the regression model backed up the conscientiousness finding by revealing significant lower conscientiousness scores in the e-coach agreeing group compared to the declining group). We see the findings in light of many studies showing that low conscientiousness goes along with higher IUD tendencies in diverse areas (Lachmann et al., 2019, Marengo et al., 2020, Montag et al., 2021) and in contrast high conscientiousness might be a buffer against IUD and therefore these persons are less likely to seek help (because it is less needed). The group choosing not to use the e-coach not only had the highest conscientiousness and lowest IUD tendencies, but also the lowest sofalizing tendencies on the facet of compensation (again this was backed up by the regression model showing a significant difference in the “sofalizing social compensation” scores between the agreeing and declining groups). Hence, these persons are less likely to cope with their loneliness with social media use (Tosuntaş et al., 2020). Putatively having other coping strategies lowers the IUD tendencies and makes them less prone to use an e-coach.

Further, we observed that the group agreeing to use the e-coach while also providing all necessary information to onboard was characterized by oldest age (but in the regression model only the contrast involving the declining group vs. neither agreeing/disagreeing group was significant). Perhaps this group is more mature in deciding to find help for one’s own problem with the Internet use or is more aware of dysfunctional Internet use. Gender did not play a role regarding group differences. The group choosing to use the e-coach also was associated with the lowest state FoMO scores. Hence, persons with lower FoMO tendencies might find it easier to choose an e-coach, because reducing online time might be easier for them, as they have less of an urge to monitor what others are doing (fittingly with our findings, it has also been observed that lower age goes along with higher FoMO tendencies; Rozgonjuk et al., 2021). It needs to be mentioned that the findings have not been hypothesized before and therefore need to be seen as exploratory. Moreover, not all contrasts in Table 1 and in parts summed up in this discussion are significant and some of the observations reported here are merely descriptive. In general, observed effect sizes were very small and one can discuss if the differences are meaningful at all (perhaps with the exception of age). Against the background of the small effect sizes, other variables must play a larger role in explaining who on-boards to use an e-coach app and those who decline. Likely this has to do with privacy reasons (Montag et al., 2020), but also other variables such as technology self-efficacy might be relevant (Montag et al., 2023), but have not been covered in this work. Interestingly, we found no differences in the agreement of using the e-coach with respect to sex. Nevertheless, sex differences might be present in the prevalence of specific IUDs with different related severities of the disorder or impairment in daily life, which in turn could be related to the willingness to use help. In addition, help-seeking for IUD in general as well the occurrence of comorbid conditions could be sex-related. These complex relationships cannot be disentangled by our data.

In summary, the present study was only able to observe few differences in several variables shedding light on why some people use an e-coach or not in the realm of IUD. The present study has several limitations. This study was not designed to test the specific research questions addressed here. As the present analyses are supplemental evaluations, better research designs, including more fitting constructs, could have carved out differences between both groups under investigation in a more in-depth manner. The present study also did not investigate how the studied variables were associated with the duration of e-coach use (“adherence”) which might be an interesting aim for future studies. Further, other variables such as employment status, or education (which have not been analysed here) might be of relevance for understanding who uses an e-coach or not. In this analysis, we focused on psychological measures because we were interested in affective and cognitive associations with IUDs. In the future, it would also be interesting to study psychological characteristics of non-clinically elevated IUD-affected persons willing to use an e-coach. This was not possible in this work due to the design of the study. Finally, it needs to be mentioned that enrolling in the present app went along with agreeing to an informed consent that included all parts of the intervention which covered answering questionnaires daily and receiving feedback. As an optional part, participants could also agree to smartphone tracking to obtain objective insights into smartphone behavior. The intensity of engagement notified for the case of being in the intervention group could have had an impact on the type of users agreeing to be part of the present project.

Of note, the app will still need to be evaluated with respect to additional areas, but this is not part of the present research question. For instance, for such an evaluation the WHO framework called “Monitoring and Evaluating Digital Health Interventions” (https://iris.who.int/bitstream/handle/10665/252183/?sequence=1) is helpful reflecting that, e. g., attrition and feasibility have to be considered. In the present work, we have not investigated measures of the app use or attrition rates in those who started to use the e-coach. This cannot be done at this stage, because the main body of research needs to be first investigated following strict protocols. These data are part of the efficacy analysis that is undertaken independently and based on blinded data by an institute for biostatistics. With respect to feasibility, data presented here clearly speak in favor of the general principle of the intervention as well as the recruitment procedure.

We believe that the present work has its merits, as we combined real-choice behavior on an e-coach with data from the screening tool that all participants filled in before making their decision to use the e-coach. Furthermore, the study size is fairly large and the topic is unique and timely. Investigating approaches to offer help via social media and other online-activities as well as public announcements could be a valuable way to reach large target populations for indicated prevention approaches with online coaching or other offers to change behavior.

This study serves as a valuable initial starting point to better understand why some people consider an e-coach as a support to overcome problem behavior – here IUD tendencies-while others decline. It should also be mentioned that the present work stems from a study dealing with IUDs. Therefore, the insights observed here might not be transferable to e-coach offerings in other areas.

## Funding

The SCAPIT study is funded by the Innovation Fund of the Federal Joint Committee Germany (grant number 01NVF19031).

## CRediT authorship contribution statement

**Christian Montag:** Writing – original draft, Methodology, Investigation, Funding acquisition, Conceptualization. **Jon D. Elhai:** Writing – original draft, Methodology, Formal analysis. **Christopher Kannen:** Project administration, Methodology. **Anja Bischof:** Writing – review & editing. **Dominique Brandt:** Writing – review & editing. **Hannah Schmidt:** Writing – review & editing, Project administration. **Dmitri Rozgonjuk:** Writing – review & editing, Project administration. **Hans-Jürgen Rumpf:** Writing – review & editing, Project administration, Funding acquisition, Conceptualization.

## Declaration of competing interest

The authors declare the following financial interests/personal relationships which may be considered as potential competing interests: Dr. Christian Montag reports no conflict of interest. However, for reasons of transparency Dr. Montag mentions that he has received (to Ulm University and earlier University of Bonn) grants from agencies such as the German Research Foundation (DFG). Dr. Montag has performed grant reviews for several agencies; has edited journal sections and articles; has given academic lectures in clinical or scientific venues or companies; and has generated books or book chapters for publishers of mental health texts. For some of these activities he received royalties, but never from gaming or social media companies. Dr. Montag mentions that he was part of a discussion circle (Digitalität und Verantwortung: https://about.fb.com/de/news/h/gespraechskreis-digitalitaet-und-verantwortung/) debating ethical questions linked to social media, digitalization and society/democracy at Facebook. In this context, he received no salary for his activities. Finally, he mentions that he currently functions as independent scientist on the scientific advisory board of the Nymphenburg group (Munich, Germany). This activity is financially compensated. Moreover, he is on the scientific advisory board of Applied Cognition (Redwood City, CA, USA), an activity which is also compensated. Dr. Jon D. Elhai notes that he receives royalties for several books published on posttraumatic stress disorder (PTSD); is a paid, full-time faculty member at the University of Toledo; occasionally serves as a paid expert witness on PTSD legal cases; and receives grant research funding from the U.S. National Institutes of Health. The remaining authors have nothing to report. The remaining authors have nothing to report. Dr. Rozgonjuk was working at Ulm University at the time of conducting this study. He now works in the private sector, and his contribution to this paper was made independently during his free time.

## Data Availability

Data will be made available on request.
