# Supplementary material for: Insights into psychological characteristics of persons (not) agreeing to use an e-coach-application to reduce elevated Internet Use Disorder tendencies
Source: Addict Behav Rep. 2024 Sep 26;20:100564. doi: 10.1016/j.abrep.2024.100564 (PMC11740796; doi:10.1016/j.abrep.2024.100564)
Supplement: Supplementary Data 1 [file mmc1.docx]

**Supplementary Table S1**. Multinomial logistic regression results using the primary continuous variables as predictor variables in classifying participants in one of the three groups indicated compared to the Not agreeing (reference) group (with unstandardized logistic regression coefficients, standard errors, and odds ratios).

| **Compared to the Not agreeing**  **Group:** | **Agreeing**  ***B* (SE), OR** | **Agreeing but not providing details**  ***B* (SE), OR** | **Neither agreeing nor disagreeing**  ***B* (SE), OR** |
| --- | --- | --- | --- |
|  |  |  |  |
| FoMO Trait | -.04 (.02), .96 | -.04 (.03), .97 | -.02 (.02), .98 |
| FoMO State | -.03 (.02), .97 | .00 (.02), 1.00 | .01 (.02), 1.01 |
| PSS | -.01 (.06), .99 | .08 (.08), 1.08 | .03 (.06), 1.03 |
| SWLS | .00 (.02), 1.00 | -.02 (.03), .98 | -.02 (.02), .98 |
| Sof-Online Displacement | -.03 (.03), .97 | -.02 (.03), .98 | -.03 (.03), .97 |
| Sof-Social Comp | .07 (.02), 1.07** | .04 (.03), 1.04 | .07 (.02),1.07** |
| CIUS | .03 (.01), 1.03* | .02 (.02), 1.02 | -.01 (.01), .99 |
| Extraversion | .02 (.03), 1.02 | .02 (.04), 1.02 | .04 (.03), 1.04 |
| Agreeableness | .00 (.04), 1.00 | .07 (.05), 1.07 | .05 (.04), 1.05 |
| Conscientiousness | -.11(.03), .89** | -.02 (.04), .98 | -.06 (.03), .94* |
| Neuroticism | .01 (.03), 1.01 | .00 (.04), 1.00 | .03 (.03), 1.03 |
| Intellect | .03 (.05), 1.03 | -.06 (.06), .94 | -.04 (.05), .96 |
| MHI-5 | -.02 (.03), .98 | -.02 (.04), .98 | .03 (.03), 1.03 |
| Age | .02 (.01), 1.02* | -.01 (.01), .99 | -.02 (.01), .98** |

Note*.* PSS = Perceived Stress Scale; SWLS = Satisfaction with Life Scale; Sof-Online Displacement = Sofalising Online Displacement; Sof-Soc Comp = Sofalizing Social Compensation; CIUS = Compulsive Internet Use Scale; MHI-5 = Mental Health Inventory-5 Scale.

* p < . 05, ** p < .01, *** p < .001
